# DO LIFE SATISFACTION AND LEVEL OF OPTIMISM IMPACT SUCCESSFUL AGING IN MID AND LATE LIFE?

**DOI:** 10.1093/geroni/igae098.2886

**Published:** 2024-12-31

**Authors:** Kallol Kumar Bhattacharyya, Victor Molinari, Debasree Das Gupta, Gizem Hueluer

**Affiliations:** Utah State University, Logan, Utah, United States; University of South Florida, Tampa, Florida, United States; Utah State University, Logan, Utah, United States; University of Bonn, Bonn, Nordrhein-Westfalen, Germany

## Abstract

Earlier theoretical frameworks of successful aging (SA) included either objective or subjective evaluations or both. Guided by the Rowe and Kahn model, the current study expands prior work by examining the association of objective and subjective components of SA with individuals’ life satisfaction and level of optimism across adulthood. Data were from waves 2 (2004-05) and 3 (2013-14) of the Midlife in the United States (MIDUS) study. To obtain a multidimensional SA score, we considered successful agers as those with a higher score in cognition, with minimal difficulties in performing physical activities, maintaining active engagement with life, and free of multiple chronic conditions. We used structural equation modeling to examine whether subjective life satisfaction (wave 2) has any effect on a composite measure of SA (wave 3) while controlling for baseline sociodemographic and health factors; we also examined the reverse directional association. Further, we also examined the mediation effects of level of optimism in the above associations. A total of 2,040 individuals (Mage=64±11; women 56%) were included. After controlling for covariates, findings revealed that prior life satisfaction has a positive direct effect (b=0.094; SE=0.019; p< 0.001) on SA; also, this effect is bidirectional. Further, high level of optimism mediates the association between life satisfaction and SA. While SA is often examined in relation to various risk/protective factors in late life, this study identified life satisfaction and level of optimism as having potentially positive impacts on achieving SA in middle-aged and older MIDUS participants.

